# Asian Pacific Association of Gastroenterology (APAGE) Inflammatory Bowel Disease (IBD) Working Party guidelines on IBD management during the COVID‐19 pandemic

**DOI:** 10.1002/jgh3.12362

**Published:** 2020-06-05

**Authors:** Khoon Lin Ling, Ida Hilmi, Raja Affendi Raja Ali, Rupert W L Leong, Wai Keung Leung, Siew Chien Ng, Kai Chun Wu, Min Hu Chen, Zhi Hua Ran, Tadakazu Hisamatsu, Vineet Ahuja, Govind K Makharia, Rupa Banerjee, Shu Chen Wei, Deng Chyang Wu, Pises Pisespongsa, Byong Duk Ye, Jose Sollano, Marcellus Simadibrata, Sai Wei Chuah, Choon Jin Ooi

**Affiliations:** ^1^ Mount Elizabeth Medical Centre Singapore; ^2^ Duke‐NUS Medical School Singapore; ^3^ University of Malaya Kuala Lumpur Malaysia; ^4^ The National University of Malaysia Bangi Malaysia; ^5^ Concord Hospital Sydney New South Wales Australia; ^6^ University of Hong Kong Hong Kong; ^7^ The Chinese University of Hong Kong Hong Kong; ^8^ Fourth Military Medical University Xi'an China; ^9^ Sun Yat‐Sen University Guangzhou China; ^10^ Shanghai Jiao Tong University Shanghai China; ^11^ Kyorin University School of Medicine Mitaka Japan; ^12^ All India Institute of Medical Sciences New Delhi India; ^13^ Asian Institute of Gastroenterology Hyderabad India; ^14^ National Taiwan University Hospital and College of Medicine Taipei Taiwan; ^15^ Kaohsiung Medical University Hospital Kaohsiung Taiwan; ^16^ Bumrungrad International Hospital Bangkok Thailand; ^17^ University of Ulsan College of Medicine Seoul South Korea; ^18^ University of Santo Tomas Manila Philippines; ^19^ Universitas Indonesia Jakarta Indonesia; ^20^ Gleneagles Medical Centre Singapore

**Keywords:** COVID‐19, Crohn's disease, inflammatory bowel diseases, ulcerative colitis

## Abstract

The COVID‐19 pandemic, secondary to SARS‐CoV‐2, has resulted in high mortality and morbidity worldwide. As inflammatory bowel disease (IBD) is a chronic disease, and most patients are on long‐term immunosuppressive agents, there is understandable concern, particularly in terms of therapy. In view of this, experts in IBD across the Asia Pacific region were invited to put together recommendations based on their experience and the currently available data. In general, most IBD therapies (with a few exceptions) can be continued safely, and the general consensus is that maintaining disease control should remain the main principle of management. In addition, social distancing measures and the appropriate use of personal protective equipment should be strictly adhered to. During the current pandemic, face‐to‐face clinic follow ups and non‐urgent procedures should be kept to a minimum.

## Abstract 2

This is a concerted effort by the members of the Asian Pacific Association of Gastroenterology Inflammatory Bowel Disease (IBD) Workgroup members to provide guidance on IBD management (all‐in‐one succinct review paper) during the difficult COVID‐19 pandemic.

## Introduction: COVID‐19 and the gastrointestinal tract

A novel coronavirus, SARS‐CoV‐2, which causes COVID‐19, first emerged in China in December 2019.^1^, ^2^ The virus has spread internationally, resulting in a pandemic.

Like SARS‐CoV, the natural host of SARS‐CoV‐2 is thought to be bats.^3^, ^4^ Like SARS and MERS, the other zoonotic coronaviruses in the recent past, the predominant symptoms of COVID‐19 are fever, upper respiratory symptoms, and shortness of breath.^1^, ^2^ Unlike the viruses that cause SARS and MERS, patients infected with SARS‐CoV‐2 may be infectious when they are presymptomatic or asymptomatic.^5^, ^6^, ^7^ From a public health perspective, this makes it harder to contain the spread of the virus, and many countries have resorted to asking all citizens to wear masks when out in the open. Infection risk from coronavirus droplets may be reduced by wearing surgical or cloth masks.^8^, ^9^

The other major difference between COVID‐19 and SARS and MERS is the morbidity and mortality of this disease. While mortality of COVID‐19 seems to be lower than SARS and MERS, there is huge public health concern primarily because it is highly infectious with rapid community spread. Severe COVID‐19 infection resulting in intensive care unit admission or in death correlates with increasing age and comorbidities, especially pre‐existing cardiovascular and pulmonary disease.^1^, ^2^, ^10^, ^11^ People younger than 20 years rarely develop severe COVID‐19.^12^ The majority of patients do not need supplemental oxygen. Those who do develop severe disease often deteriorate in the second week of illness.^2^, ^10^, ^11^ This is thought to be due to a cytokine storm.^13^, ^14^

Diagnosis of SARS‐CoV‐2 infection depends on swab of the nasopharynx and/or the throat and PCR for viral RNA.^15^ The sensitivity is 90% or more in the first week of illness, although this subsequently drops further in the course of infection. Tests for antibodies to SARS‐CoV‐2 using ELISA or point‐of‐care test kits are not sensitive within 14 days of symptom onset.

SARS‐CoV‐2 may affect organs other than the upper airways and the lung. Between 15 and 50% of patients may have gastrointestinal (GI) symptoms in addition to respiratory symptoms.^16^, ^17^, ^18^, ^19^ In a case series of COVID‐19 patients in China, 17% had diarrhea, 1.9% had vomiting, and 0.9% had abdominal pain.^16^ The diarrhea is often not profuse and usually occurs up to three times per day. Of patients in this series, 3% presented with only fever and GI symptoms. While this case series showed that patients with more severe COVID‐19 were more likely to have GI symptoms, this finding has not been replicated in all case series of COVID‐19 patients. The results of a meta‐analysis from 35 studies, including 6686 patients with COVID‐19, showed that the pooled prevalence of digestive system comorbidities was 4%, and the pooled prevalence of digestive symptoms was 15%, with nausea or vomiting, diarrhea, and loss of appetite being the three most common.^20^

The GI symptoms are not a nonspecific response to sepsis and result from viral infection of intestinal epithelial cells mediated through the ACE2 receptor.^17^ The virus is found in stools and may continue to be shed for another 1–2 weeks after the nasal and throat swabs have become negative.^21^, ^22^ It is not known if the viral particles found in the stools continue to be infectious.

## COVID‐19 and IBD

Inflammatory bowel diseases (IBD), such as ulcerative colitis and Crohn's disease (CD), are chronic inflammatory conditions that require long‐term treatment. There is currently no data to suggest that IBD patients on corticosteroids, immunomodulators, biologics, and small molecules are at increased risk of contracting SARS‐CoV‐2 or developing COVID‐19. Data from Chinese and European IBD centers suggest that IBD patients, including those on biological agents, are not more likely to be infected.^23^, ^24^, ^25^ Mortality among IBD patients with COVID‐19 is not higher than among the general population.

The SECURE IBD database collects data on IBD patients infected by SARS‐CoV‐2. At the time of writing, despite there being almost 2 million infected individuals in the world, fewer than 1000 patients have been reported in SECURE IBD.^26^

The IBD management principle should always be to maintain disease control during this period while preserving patient safety.

### *Social distancing*

All IBD patients should practice an appropriate degree of social distancing as advised by each country's health authority and use facial mask protection when outside. Depending on age, comorbidities, and the type of medication a patient is on, some patients will be at higher risk of poor outcome should they be infected with SARS‐CoV‐2.^27^

## Medication for patients with IBD not infected with SARS‐CoV‐2

The potential effect of IBD therapy on COVID‐19 is uncertain given that few patients have been infected to date, with no obvious detrimental effects from any single medication class.^25^, ^28^ As such, the following advice is based on theory and pooled consensus based on various gastroenterological societies. There is as yet no evidence that being on immunosuppressive therapies increases the risk of acquiring SARS‐CoV‐2 infection or of developing COVID‐19. IBD patients should not start or stop their medications without consulting their physicians.

Patients with COVID‐19 may present with only fever and GI symptoms. Therefore, the IBD physician must have a high index of suspicion, especially in patients with positive COVID‐19 contact history or those who fulfill the relevant travel history to countries with COVID‐19 outbreaks. In these cases, the physician should have a low threshold to test for SARS‐CoV‐2.

### *5‐aminosalicylic acid compounds*

Among the IBD medicines, 5‐aminosalicylic acid compounds such as mesalazine do not increase the risk of infection and should be continued.

### *Corticosteroids*

Corticosteroid use should be minimized. Tapering of corticosteroids should be considered for doses exceeding 20 mg/day of prednisolone or equivalent, guided by the disease activity. For patients who require induction, corticosteroids should have its dose tapered as quickly as disease activity permits. Alternative, topical treatment or oral budesonide (Cortiment, Budenofalk, Entocort) are considered safer alternatives.

### *Thiopurines/methotrexate*

Thiopurines (azathiopurine, mercaptopurine) may cause leukopenia, which may impair one's immunity against virus. However, patients who are already on thiopurines should continue taking the drug if they are well controlled. Likewise, it is also recommended for patients to continue with methotrexate.

### *Biological agents*

Patients already on a biological agent and in remission should continue taking the current drug. Dose reduction, if deemed necessary, might be considered in an appropriate patient who has recent documented mucosal healing upon discussion with the managing physician. A patient should not switch from an intravenous biologic (e.g. IV infliximab) to a subcutaneous biological (e.g. SC adalimumab) if there is good response to the original drug, except in extenuating circumstances when infusion centers are not available. There is currently no evidence that any class of biological agents is safer than another. Without further data, we do not recommended switching from one class of biological therapy (e.g. anti TNF agent) to another biologic (e.g. non‐anti‐TNF agent) if the patient is in remission. Additional precautions may be warranted during visits to an infusion center for intravenous infusions, such as by wearing a mask and observation of personal hygiene.

Patients who wish to stop biological agents during this time should fulfill the same criteria for stopping biological agents during nonpandemic times.

For patients who are considering new combination therapy (e.g. thiopurine and infliximab), settling for monotherapy biological agents for the initial period during the pandemic may be considered in view of increased immunosuppression with combination therapy.

In patients in whom the initiation of biological agents is considered, vedolizumab and ustekinumab may be better alternatives given their less systemic immunosuppressive activity. These newer agents seem to be less likely to develop immunogenicity and less reliant on immunomodulatory agent cotherapy. Vedolizumab and ustekinumab may also be preferred in higher‐risk elderly individuals.

### *Tofacitinib/JAK* *inhibitors*

There are no data to ascertain if tofacitinb or other JAK inhibitors increase the risk of SARS‐CoV‐2 infection and COVID‐19. There is laboratory evidence that JAK inhibitors may reduce interferon alpha production, a cytokine important for antiviral immunity. However, JAK inhibitors, for example, baricitinib, may mitigate the cytokine storm that occurs during the hyperinflammatory phase of COVID.^29^

Patients in remission on tofacitinib should be maintained on the lowest effective dose.

### *Investigational products (clinical trials)*

Patients in clinical trials should not stop their trial medications. If these patients develop SARS‐Co‐2 infection or COVID 19, the trial medications should be interrupted and the study sponsors immediately updated. Most clinical drug trials have currently frozen the screening of new potential cases.

#### *Fecal microbiota transplantation*

SARS‐CoV‐2 can infect intestinal epithelial cells, and viral particles have been identified in stools. Fecal microbiota transplantation (FMT) should therefore be reserved for life‐threatening conditions such as severe *Clostridioides difficle* infections, ideally using samples collected prior to the COVID outbreak. FMT centers collecting stool donations after the start of the COVID‐19 pandemic should develop protocols to screen fecal material for SARS‐CoV‐2.^30^

## Exclusive enteral nutrition

Exclusive enteral nutrition is a safe and effective option to induce remission in CD without risking the development or worsening of COVID‐19. Comanagement with a dietician is advised.

## Vaccinations

Patients who are receiving immunomodulators or biological agents are recommended to receive up‐to‐date vaccinations against influenza and *Pneumonococcus*.

## Surgery and endoscopy

IBD patients should not undergo elective endoscopies if detrimental effects are not expected in such postponement. Noninvasive markers, such as serum C‐reactive protein and fecal calprotectin, can be used to assist with disease activity assessment.

Patients in whom endoscopic results will have a major impact on disease management in the short term should proceed with endoscopy in accordance with local best practices. A screening process should be undertaken to exclude SARS‐CoV‐2 infection based on local and national policies and practices. They usually include exposure history, blood or nasal/pharyngeal swab confirmatory tests, and chest computed tomography scans.^31^

The urgent IBD‐related surgeries generally cannot be postponed without adverse consequences to the patient. These should not be deferred.

## Follow up of IBD patients

Where there is significant community transmission of SARS‐CoV‐2, doctors may consider teleconsultation with IBD patients in place of face‐to‐face clinic consultations.

In addition, in countries where IBD drugs are not usually available in community pharmacies, a mechanism may have to be in place to deliver drugs to the patients at their homes.

For patients with poor or suboptimal control of disease, a face‐to‐face consult should be considered on a case‐by‐case basis, depending on the severity of community transmission of SARS‐CoV‐2.

The usual indications for hospitalizations of IBD patients should prevail.

## Treatment of IBD patients infected with SARS‐CoV‐2

### *Patients with* *SARS‐CoV‐2 infection who are asymptomatic or have minimal symptoms without pneumonia*

IBD patients in remission with no or minimal symptoms without pneumonia should stop immunomodulators (thiopurines, methotrexate) and JAK inhibitors for the first 2 weeks of infection.^32^ The next dose of maintenance biological agents should be delayed until after the first 2 weeks of diagnosis of SARS‐CoV‐2 infection. Corticosteroids should be tapered as quickly as possible. If they have not developed pneumonia and do not require oxygen by the third week of illness, the patient may resume immunomodulators and biological agents.

IBD patients not in remission should not have their active treatment reduced. Instead, patients with active IBD should be started on the most effective IBD therapy except for azathioprine, methotrexate, and tofacitinib.

### *Patients with* *SARS‐CoV‐2 infection with pneumonia*

IBD patients in remission who develop COVID‐19 pneumonia should stop thiopurines, methotrexate, and tofacitinib and postpone receiving maintenance doses of biological agents until clearance of the virus.^32^ Patients on corticosteroids should taper the dose unless they risk hypoadrenocortical responses in the setting of sepsis.

Patients with moderate to severe COVID‐19 may be offered novel therapy for treatment of the infection in a clinical trial setting. In addition to antiviral agents (e.g. remdesivir), anti‐TNFα, anti‐IL‐6 (e.g. tocilizumab), and JAK Inhibitors (e.g. baricitinib) are also undergoing clinical trials.^33^, ^34^, ^35^ Gastroenterologists are already familiar with anti‐TNFα in the treatment of IBD. There are data suggesting that anti‐IL‐6 inhibitors are useful in CD as well.^36^ It may therefore be reasonable to use anti‐TNFα and anti‐IL‐6 in patients with both active CD and COVID‐19, should the need arise.

Patients who interrupted their IBD medications and subsequently recover from COVID‐19 can restart their medications once they are confirmed negative for SARS‐CoV‐2.

## Conclusion

In summary, there is no evidence that the current therapies for IBD increase the risk of SARS‐CoV‐2 infection or the development of severe COVID‐19. Some therapies, such as anti‐TNFα, anti‐IL‐6, and JAK inhibitor, may conversely have a beneficial role in ameliorating severe COVID‐19 disease, although this has yet to be proven. The main take‐home message is that IBD patients should not discontinue their current therapy during the COVID‐19 pandemic and that most therapies can be safely initiated if indicated.
